# Complement and coagulation cascades pathway-related signature as a predictor of immunotherapy in metastatic urothelial cancer

**DOI:** 10.18632/aging.205022

**Published:** 2023-09-24

**Authors:** Zheng Gong, Yuming He, Xiao Mi, Chengcheng Li, Xiaoran Sun, Guoqiang Wang, Leo Li, Yusheng Han, Chunwei Xu, Wenxian Wang, Shangli Cai, Liang Wang, Zhongyuan Liu

**Affiliations:** 1 Department of Urology, Shengjing Hospital of China Medical University, Shenyang 110001, China; 2 Burning Rock Biotech, Guangzhou 510300, China; 3 Institute of Basic Medicine and Cancer (IBMC), Chinese Academy of Sciences, Hangzhou 310022, China; 4 Department of Clinical Trial, The Cancer Hospital of the University of Chinese Academy of Sciences (Zhejiang Cancer Hospital), Hangzhou 310022, China; 5 The First Affiliated Hospital of Dalian Medical University, Dalian 116011, China

**Keywords:** urothelial cancer, immune checkpoint inhibitor, complement and coagulation cascades, predictive biomarker

## Abstract

Background: Immune checkpoint inhibitors (ICIs) have shown efficacy in patients with metastatic urothelial cancer (mUC), however, only a small subset of patients could benefit from ICIs. Identifying predictive biomarkers of ICIs in patients with mUC is clinical meaningful for patient stratification and administration.

Methods: Clinical and transcriptomic data of mUC patients treated with ICIs from mUC cohort (IMvigor210 study) was utilized to explore the predictive biomarkers. LASSO Cox regression was performed to construct a predictive model. The predictive model was trained and tested in the mUC cohort, and then exploratively tested in clear cell renal cell carcinoma (ccRCC) and melanoma cohorts in which patients also received ICIs regimens.

Results: The differentially expressed genes (DEGs) in complement and coagulation cascades pathway (CCCP) were mainly enriched in non-responders of ICIs in the mUC cohort. A CCCP risk score was constructed based on the DEGs in CCCP. Patients with a low-risk score were more responsive to ICIs and had better overall survival (OS) than those with a high-risk score in the training set (HR, 0.38; 95%CI, 0.27-0.53, P<0.001) and the test set (HR, 0.34; 95%CI, 0.17-0.71, P=0.003). The association between the CCCP risk score and OS remained significant in the multivariable cox regression by adjusting PD-L1 expression and TMB (P<0.05). In addition, there was no difference for OS in the bladder cancer patients without ICIs (TCGA-BLCA cohort, HR, 0.76, 95%CI, 0.49-1.18, P=0.22), suggesting a predictive but not prognostic effect of the risk score. For the exploratory analysis, consistent results were observed that low-risk group showed superior OS in ccRCC cohort (HR, 0.52, 95%CI, 0.37-0.75, P<0.001) and melanoma cohort (HR, 0.27, 95%CI, 0.12-0.62, P=0.001).

Conclusions: Our study showed that the CCCP risk score is an independent biomarker that predicts the efficacy of ICIs in mUC patients. The patients with a low-risk score tend to have a better response to ICIs and a longer life time probably due to the immune-activated TME. Further studies are needed to validate the clinical utility of the seven-gene signature.

## INTRODUCTION

Urothelial carcinoma (UC) is a common type of cancer, which is derived from the pseudostratified epithelium. UC is the 10th most commonly diagnosed cancer worldwide, with more than 573,000 new cases and 213,000 deaths in 2020 [1]. Bladder cancer is the most common malignancy of UC, accounting for 90% to 95% [2]. Although the advance of immune checkpoint inhibitors (ICIs) has led to substantial improvements in outcomes for patients with metastatic UC (mUC), the response rate of ICIs is only around 20% and the 5-year survival rate is only 15% [3]. These concerns have prompted studies to identify the mUC patients who are most likely to benefit from ICIs.

Previous studies have identified several predictive biomarkers for ICIs treatment in mUC, including programmed death-ligand 1 (PD-L1) expression [4], CD8^+^ T cell [5, 6], tumor mutational burden (TMB) [7, 8], microsatellite instability (MSI) [5], and tumor-infiltrating lymphocytes (TILs) [9, 10]. Despite these advances, there are still a majority of mUC patients showing unresponsiveness to ICIs. Therefore, the identification of more convenient and reliable biomarkers beyond TMB and PD-L1 expression for the prediction of ICIs benefits are needed for clinical practice.

The coagulation and complement cascades pathway (CCCP) exert multiple positive or negative effects on tumorigenesis and mediate the components of the tumor microenvironment (TME) [11–13]. Complement, an essential part of innate immunity, converges at the cleavage of C3 and C5 upon activation and leads to the release of the anaphylatoxins C3a, C4a, and C5a, thereby leading to the lysis of target cells by the membrane attack complex [14]. Monoclonal antibody (mAb)–based cancer immunotherapy relies on the two-pronged capacity of mAbs to halt oncogenic signaling and tumor cell growth and to simultaneously fix complement on the surface of the targeted tumor cells, thereby eliciting complement-dependent cytotoxicity (CDC) [15–17]. Previously, many studies of preclinical models in lung, colon, and liver cancers have indicated complement-derived inflammatory mediators, such as C5a, together with PD-1 blockade markedly reduced tumor growth and metastasis and lead to prolonged survival via enhancing antitumor CD8^+^ T cell responses [18–20]. Moreover, cancer cells can exploit the CCCP to shape the tumor microenvironment (TME), thus impacting the efficacy of ICIs [21, 22]. For example, Markiewski et al. found the production of C5a in TME recruited myeloid-derived suppressor cells (MDSCs) to restrain the antitumor effect of CD8^+^ T cell and thus promoting tumor growth in cervical cancer mouse model [23]. Tumor-associated macrophages (TAM) has been hijacked by RCC tumor cells to produce C1q and then activated the complement signal and the expression of C1q was associated with an exhausted T cell phenotype and poor clinical outcome [24]. Corrales et el. have reported that lung cancer cells were capable of producing C5a, which contributed to the recruitment of MDSCs and generation of an immunosuppressive microenvironment in lung cancer [25]. Altogether, these studies suggest that CCCP may play an important role in shaping TME to impact immunotherapeutic efficacy and cancer progression. However, the role of CCCP in ICIs treatment has not been fully studied.

In the present study, we aimed to explore a predictive biomarker for immunotherapeutic responsiveness in mUC. We identified that CCCP was associated with the efficacy of anti-PD-1/PD-L1 treatment in patients with mUC. Based on the CCCP, we developed and validated a seven-gene signature as an independent predictive biomarker of ICIs.

## MATERIALS AND METHODS

### Data source and study design

The clinical and mRNA gene expression data of 298 mUC patients from IMvigor210 study (mUC cohort) are publicly available in the R package “IMvigor210CoreBiologies” which was downloaded from website http://research-pub.gene.com/IMvigor210CoreBiologies/ [26]. The Cancer Genome Atlas (TCGA)-BLCA dataset is publicly available in the TCGA database, which comprises 409 bladder cancer samples with gene expression and 401 patients with survival and clinical characteristics. The RNA-seq data in the mUC cohort and TCGA-BLCA cohort was transformed into transcripts per million (TPM) data by R package “GeoTcgaData”, and then processed by Log2 transformation before analysis. The gene expression profiles of advanced clear cell renal cell carcinoma cohort (ccRCC cohort) were acquired from published literature, which comprises of 181 patients who received Nivolumab [27], and no other data processing was performed for subsequent analysis. A melanoma dataset (melanoma cohort) including 40 patients with metastatic melanoma, was obtained from cBioPortal [28]. The expression data in the melanoma cohort was normalized by Z-score transformation. Relevant clinical data of these bladder and other carcinoma samples are summarized in Supplementary Table 1.

### Identification of differentially expressed genes (DEGs) and enrichment analysis

Tumor were assessed according to the Response Evaluation Criteria in Solid Tumors (RECIST) version 1.1 [29]. Responders were defined as patients with complete response (CR) and partial response (PR) after ICIs treatment, contrary, non-responders were defined as patients with stable disease (SD) and progressive disease (PD). DEGs analysis was performed between responders and non-responders by R package “DESeq2” [30] with cut-off parameters of fold change > 1.5 (|log2FC|>0.5849625) and P-value < 0.05. R package “clusterProfiler” [31] was used to perform pathway enrichment analysis of DEGs with Kyoto Encyclopedia of Genes and Genomes (KEGG). The threshold was set as false discovery rate (FDR) < 0.05, and q-value < 0.2. To further investigate the enriched pathway, single sample gene set enrichment analysis (ssGSEA) [32] was performed to assess the enrich level of pathway (ssGSEA score) for each sample with R package “GSEABase”.

### Construction of the CCCP risk score

In order to construct a predictive signature of ICIs in mUC, 69 candidate genes in CCCP were obtained from the Molecular Signatures Database (MSigDB) [33] (Supplementary Table 2). The core genes were selected by the least absolute shrinkage and selection operator (LASSO) regression analysis. The mUC cohort were divided into training and validation cohort randomly, and repeated 1000 times. We summarized the results of Lasso regression and picked genes with frequencies greater or equal to 300 in the analysis. Lasso regression analysis was via R package “glmnet” with parameters nlambda=100, alpha=1, and family=cox. The above selected genes were then examined by multivariable Cox regression. The CCCP risk model scores were calculated by the formula: $CCCP\;risk\;score=\sum_{i}^{n}\left(Exp_{i}*Coef_{i}\right)$, where Exp_i_ and Coef_i_ represents the expression value and the cox coefficient of the selected genes, n is equal to the number of selected genes.

### Estimates of tumor infiltrating leukocytes

Immune cell infiltrations were evaluated by Estimating Relative Subsets of RNA Transcripts (CIBERSORT) based on the gene expression data [34, 35]. CIBERSORT gene signature matrix, termed LM22, contains 547 genes and distinguishes 22 human hematopoietic cell phenotypes, including seven T cell types, naive and memory B cells, plasma cells, NK cells, and myeloid subsets. We analyzed the proportions of immune cells in mUC, TCGA-BLCA, ccRCC, and melanoma cohorts to explore the patterns of TILs in different groups with the number of permutations set at 100.

### Statistical analysis

Statistical tests were performed using R software, version 4.0.1 (R Foundation for Statistical Computing Vienna, Austria). Differences in overall survival (OS) between groups were compared using Kaplan-Meier curves, with P-values calculated via the log-rank test using the R package “survival”. Hazard’s ratio (HR) was determined by univariable Cox proportional regression. Parameters with P-value < 0.05 in the univariable Cox proportional regression were subjected to multivariable Cox regression. Receiver operating characteristic (ROC) curve was drawn and the area under curve (AUC) was used to show the predictive ability of the risk model. All reported P-values were two-sided and P < 0.05 was considered statistically significant.

### Data availability statement

The datasets used and/or analyzed during the current study are available from the corresponding author on reasonable request.

## RESULTS

### Identification of DEGs between responders and non-responders of ICIs regimen

**Figure 1:**
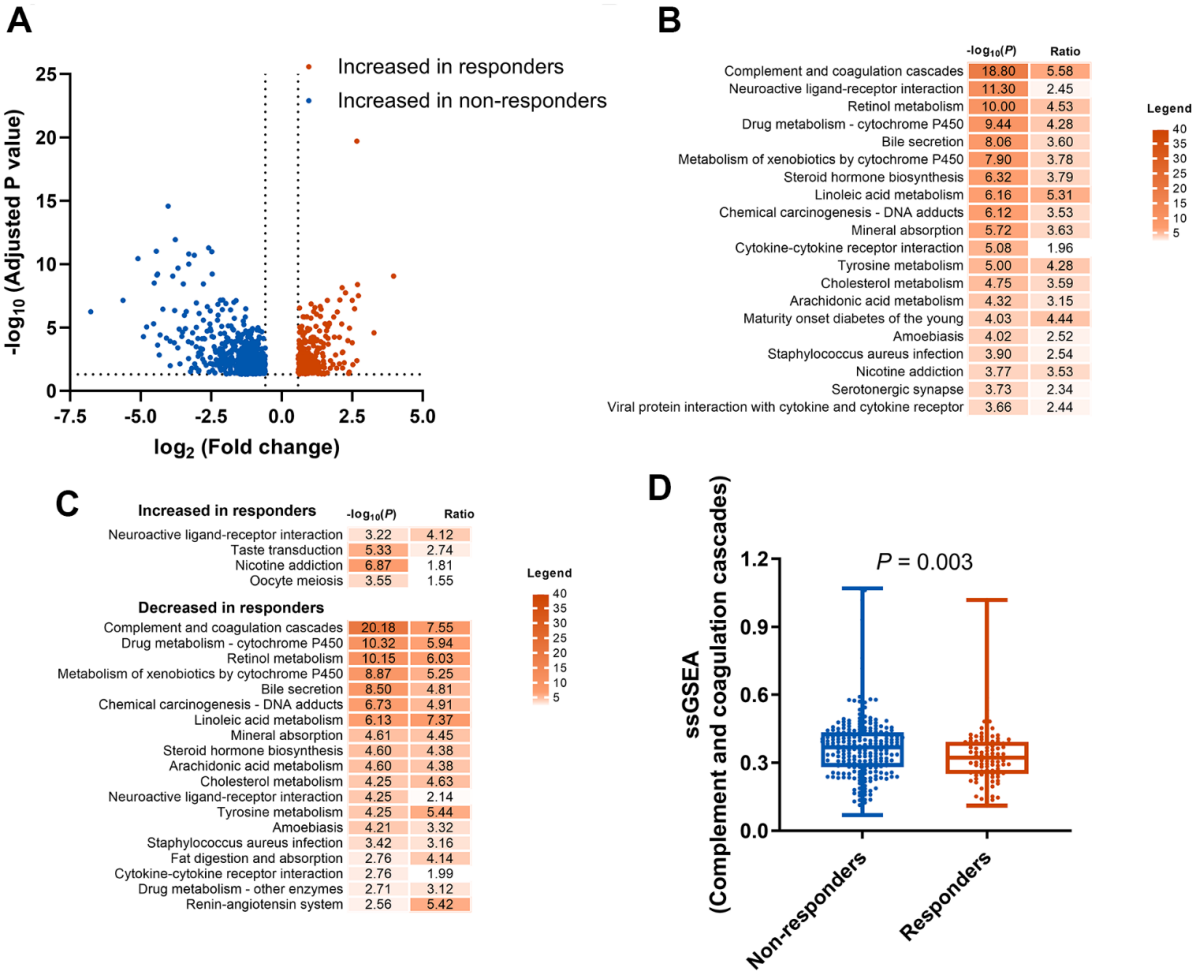
**Identification and enrichment analysis of DEGs.** (**A**) DEGs between responders (CR or PR) and non-responders (SD or PD) groups. (**B**) KEGG pathway enrichment analysis of the 1613 DEGs. (**C**) KEGG pathway enrichment analysis of the increased and decreased genes in responders. (**D**) Comparison of complement and coagulation cascades pathway score generated by ssGSEA between responders and non-responders. DEGs, differentially expressed genes; KEGG, Kyoto Encyclopedia of Genes and Genomes; CR, complete response; PR, partial response SD, stable disease; PD, progressive disease; ssGSEA, single sample gene set enrichment analysis.

In order to identify potential predictive biomarkers of ICIs, we explored the DEGs between responders and non-responders to ICIs in the mUC cohort. In total, 1,613 DEGs were identified with 1,080 genes upregulated and 533 genes downregulated in responders group (Figure 1A). KEGG enrichment analysis for the 1,613 genes identified 28 significant different pathways (P<0.05) between responders and non-responders of ICIs (Figure 1B and Supplementary Table 3), including complement and coagulation cascades pathway, which was also enriched in the downregulated genes of responders (Figure 1C). To further study the association between CCCP and response to ICIs, ssGSEA algorithm was performed to calculate the CCCP enrichment score, which represents the degree of absolute enrichment of CCCP in each patient. As a result, the CCCP score was significantly lower in responders compared to non-responders (P = 0.003, Figure 1D), suggesting that the majority of CCCP-related genes were downregulated in the responders of ICIs in mUC. Taken together, these results suggested that CCCP might be associated with the immunotherapeutic responsiveness in patients with mUC.

### Construction of a CCCP signature that predicts efficacy for ICIs

**Figure 2:**
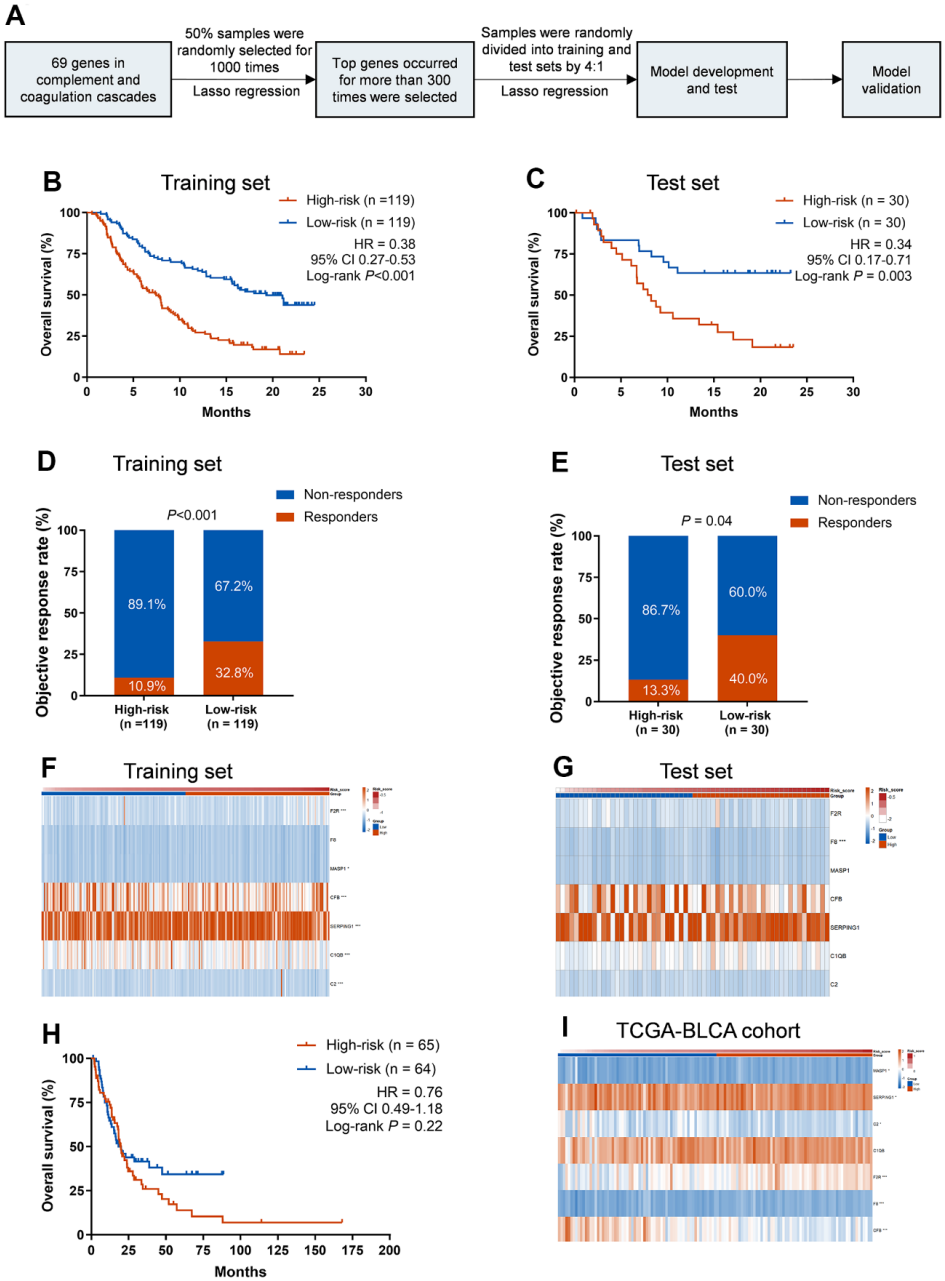
**Training and validation of the CCCP risk score in mUC and TCGA-BLCA cohort.** (**A**) Workflow for the construction of the CCCP risk model. (**B**, **C**) Kaplan-Meier curves of OS comparing patients with high- and low-risk in the training (**B**) and test sets (**C**). (**D**, **E**) Comparison of objective response rate between patients with high- and low-risk in the training (**D**) and test sets (**E**). (**F**, **G**) Heatmaps depicting the expression of the seven core genes from CCCP in patients with high- and low-risk in the training (**F**) and test sets (**G**). (**H**) Kaplan-Meier curves of OS comparing patients with high- and low-risk in TCGA-BLCA cohort (**I**) Heatmap depicting the expression of the seven core genes from CCCP in patients with high- and low-risk in TCGA-BLCA cohort. CCCP, complement and coagulation cascades pathway; mUC, metastatic urothelial carcinoma; OS, overall survival.

To further demonstrate the role of CCCP in the efficacy of ICIs, a total of 69 genes that regulate or mediate CCCP were collected as candidate genes from MSigDB. To specifically identify the core genes which predict response to ICIs, a predictive risk score was constructed based on the expression of the candidate genes using the LASSO regression analysis (Figure 2A). Seven core genes including *C2, CFB, C1QB, SERPING1, MASP1, F8*, and *F2R*, with highest frequency of features occurrence in LASSO analysis, were selected as robust markers for further study. Expression of all the seven genes was associated with the efficacy of ICIs in patients with mUC (Log-rank test, P<0.05, Supplementary Figure 1).

Then we constructed a CCCP risk model using a data-splitting strategy (Supplementary Figure 2) that randomly separate the mUC cohort into training and test cohorts with a ratio of 4:1. This CCCP risk model was to comprehensively investigate the association between the seven genes in CCCP and OS in mUC patients (Figure 2A). We repeated this process for 1,000 times and the risk score constructed in the training set was associated with OS in the test set for 857 times (Supplementary Table 4), indicating the robustness of the model.

To further demonstrate the association between the CCCP risk score and OS, patients were divided into low-risk group and high-risk group based on the median cutoff value. Patients in low-risk group had a better OS than those in high-risk group in the training set (median OS, 19.3 vs 7.5 months; HR, 0.38; 95% CI, 0.27-0.53, P<0.001, Figure 2B). Consistent results were observed that low-risk group exhibited a superior OS in the test set (median OS, not reached vs 8.1 months; HR, 0.34; 95% CI, 0.17-0.71, P = 0.003, Figure 2C). The objective response rate (ORR) was also significantly higher in low-risk patients than that of high-risk patients in both training and test sets (training set, 32.8% vs 10.9%, P<0.001, Figure 2D; test set, 40.0% vs 13.3%, P = 0.04, Figure 2E). Besides, the AUC of response predictive ability reached 0.714 (95% CI, 0.639-0.789) and 0.656 (95% CI, 0.514-0.798) in the training set and test set, respectively (Supplementary Figure 3). The different expression of the seven genes included in the CCCP risk score in the training and test sets are depicted in Figure 2F, 2G and Supplementary Figure 4.

To explore whether the CCCP risk score was an independent predictive biomarker for ICIs, we performed univariable and multivariable Cox regression analysis with the CCCP risk score and clinical characteristics including TMB, sex, intravesical BCG administered, ECOG, platinum-contained regimens history, and PD-L1 expression. Variables with P-value <0.05 in univariable Cox regression were further included in the multivariable Cox regression. The association between the CCCP risk score and OS remained significant in both training and test sets after adjusting TMB and PD-L1 expression (training set: HR, 0.43, 95% CI 0.27-4.53, P<0.001; test set: HR, 0.39, 95% CI 0.17-0.90, P = 0.03, Table 1). Altogether, our results suggested that the CCCP risk score might serve as an independent biomarker for predicting response to ICIs in mUC.

**Table 1:** Univariable and multivariable Cox analysis analyses of OS in mUC patients treated with ICIs.

**Variables**	**Training set**							**Test set**						
	**Univariable analysis**				**Multivariable analysis**			**Univariable analysis**				**Multivariable analysis**		
	**HR**	**95% CI**	**P**		**HR**	**95% CI**	**P**	**HR**	**95% CI**	**P**		**HR**	**95% CI**	**P**
Risk score														
Low vs high	0.38	0.27-0.53	<0.001		0.43	0.28-0.65	<0.001	0.34	0.17-0.71	0.004		0.39	0.17-0.90	0.03
TMB														
>10 muts/mb vs ≤ 10 muts/mb	0.5	0.33-0.76	0.001		0.59	0.39-0.90	0.01	0.81	0.38-1.76	0.6		0.98	0.44-2.17	0.95
Sex														
Male vs female	0.81	0.56-1.19	0.28					0.89	0.41-1.92	0.77				
ECOG														
2 vs 0-1	0.94	0.44-2.00	0.87					NA	NA	NA				
Received platinum														
Yes vs no	1.37	0.91-2.06	0.14					1.37	0.56-3.31	0.49				
Intravesical BCG administered														
Yes vs no	0.87	0.60-1.26	0.46					1.29	0.56-2.99	0.55				
PD-L1														
TC2 vs TC0-1	0.92	0.59-1.43	0.71					1.21	0.47-3.12	0.7				
IC2 vs IC0-1	0.52	0.37-0.75	<0.001		0.8	0.52-1.23	0.31	0.81	0.40-1.68	0.58		0.45	0.20-1.04	0.06

Abbreviations: OS, overall survival; TMB, tumor mutation burden; ECOG, Eastern Cooperative Oncology Group; BCG, bacillus Calmette-Guerin, HR, Hazard Ratio; 95% CI, 95% confidence interval.

To explore whether the CCCP risk score could affect the outcome of the mUC patients without immunotherapy, we further selected stage IV bladder cancer patients who mainly accepted chemotherapy from TCGA-BLCA cohort and conducted the same analysis. There was no difference in OS between high- and low-risk group in stage IV bladder cancer patients (median OS, 18.1 vs 19.8 months; HR, 0.76; 95% CI 0.49-1.18, P = 0.22, Figure 2H), suggesting that CCCP risk score might serve as a predictive biomarker of OS benefit from immunotherapy rather than chemotherapy. Moreover, except for C1QB, higher expression of the other six genes were observed in the low-risk group (P<0.05, Figure 2I). Overall, these findings demonstrated that the association between CCCP risk score and OS of patients was most likely derived from the different response to ICIs.

### Association between the CCCP risk score and immune cell infiltrates

**Figure 3:**
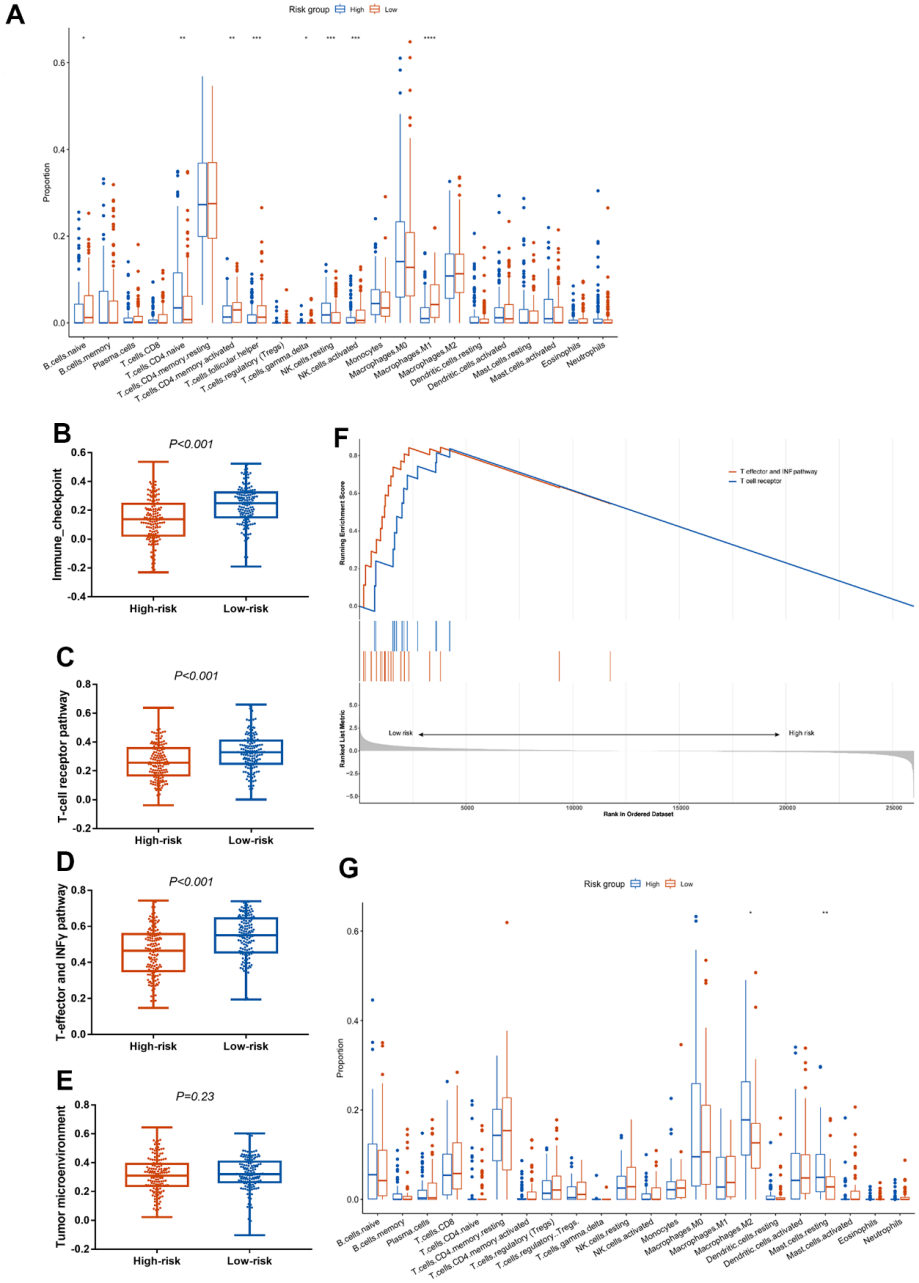
**Association between the CCCP risk score and immune microenvironment in mUC and TCGA-BLCA cohort.** (**A**) Comparison of immune cell infiltrations between patients with high- and low- risk score in mUC cohort. (**B**–**E**) Comparison of ssGSEA scores in immune checkpoint (**B**), T-cell receptor (**C**), T-effector and INF-γ (**D**) and tumor microenvironment (**E**) associated pathway between patients with high- and low-risk score. (**F**) GSEA enrichment analysis of T-cell receptor pathway and T-effector and INF-γ pathway in patients with high- and low-risk score. (**G**) Comparison of immune cell infiltrations between patients with high- and low- risk score in TCGA-BLCA cohort. mUC, metastatic urothelial carcinoma; CCCP, complement and coagulation cascades pathway; ssGSEA, single sample gene set enrichment analysis; GSEA, gene set enrichment analysis.

To further investigate the association between the CCCP risk score and immune characteristics, we compared the immune cell infiltrates between low-risk and high-risk groups using CIBERSORT algorithm [34]. We found that macrophages M1, activated NK cells, activated CD4^+^ memory T cells, and T follicular helper cells (Tfh) were significantly higher in low-risk group in mUC cohort, while naive CD4^+^ T cells and resting NK cells were significantly higher in high-risk group (P<0.01, Figure 3A). In addition, correlation analysis of these tumor infiltrating immune cells and CCCP risk score revealed that the infiltration level of macrophages M1 (P<0.001), activated CD4 ^+^ memory T cells (P = 0.006), Tfh (P = 0.02), and activated NK cells (P = 0.04) were negatively correlated to the risk score, while naive CD4^+^ T cells (P < 0.001) and resting NK cells (P = 0.04) were positively correlated with the risk score (Supplementary Figure 5), suggesting that CCCP risk score was correlated with the immune activity in TME. The correlation analysis between immune cell infiltrations and the individual genes included in the CCCP risk score were illustrated in Supplementary Figure 6. The proportion of macrophages M1 was positively correlated with the expression of C2, *C1QB, SERPING1, CFB*, and *F2R*. The proportion of activated CD4^+^ memory T cells was positively correlated with the expression of *C2, C1QB, SERPING1* and *CFB.* Moreover, the proportion of CD8^+^ T cell and activated NK cell were positively correlated with *C2* and *C1QB* expression. By contrast, the proportion of pro-tumor immune cells such as naive CD4^+^ T cells, activated dendritic cells, and resting NK cells were negatively correlated with the expression of several signature genes including *C2, F2R,* and *C1QB*. Besides, ssGSEA analysis were used to distinguish the immune characteristics between high- and low-risk groups. As a result, the activity of immune checkpoint, T cell receptor, and T-effector and IFN-γ pathway were significantly higher in low-risk group than that in high-risk group (Figure 3B–3E) by ssGSEA analysis. Consistent results were observed that T-effector and IFN-γ pathway and T-cell receptor pathway were enriched in low-risk group (Figure 3F).

We also analyzed immune cell infiltrations in the TCGA-BLCA cohort. Similar to the result of mUC cohort, macrophages M2 and resting mast cells, which representing immunosuppressive environment [36], were higher in high-risk group compared with that in low-risk group in TCGA-BLCA cohort (Figure 3G). In summary, these results indicated that the composition of immune cells in the TME may favor an immunosuppressive environment that promotes tumor progression in high-risk group, thus supporting the predictive utility of the CCCP risk score in predicting response to ICIs.

### Exploratory analysis of the CCCP risk score

**Figure 4:**
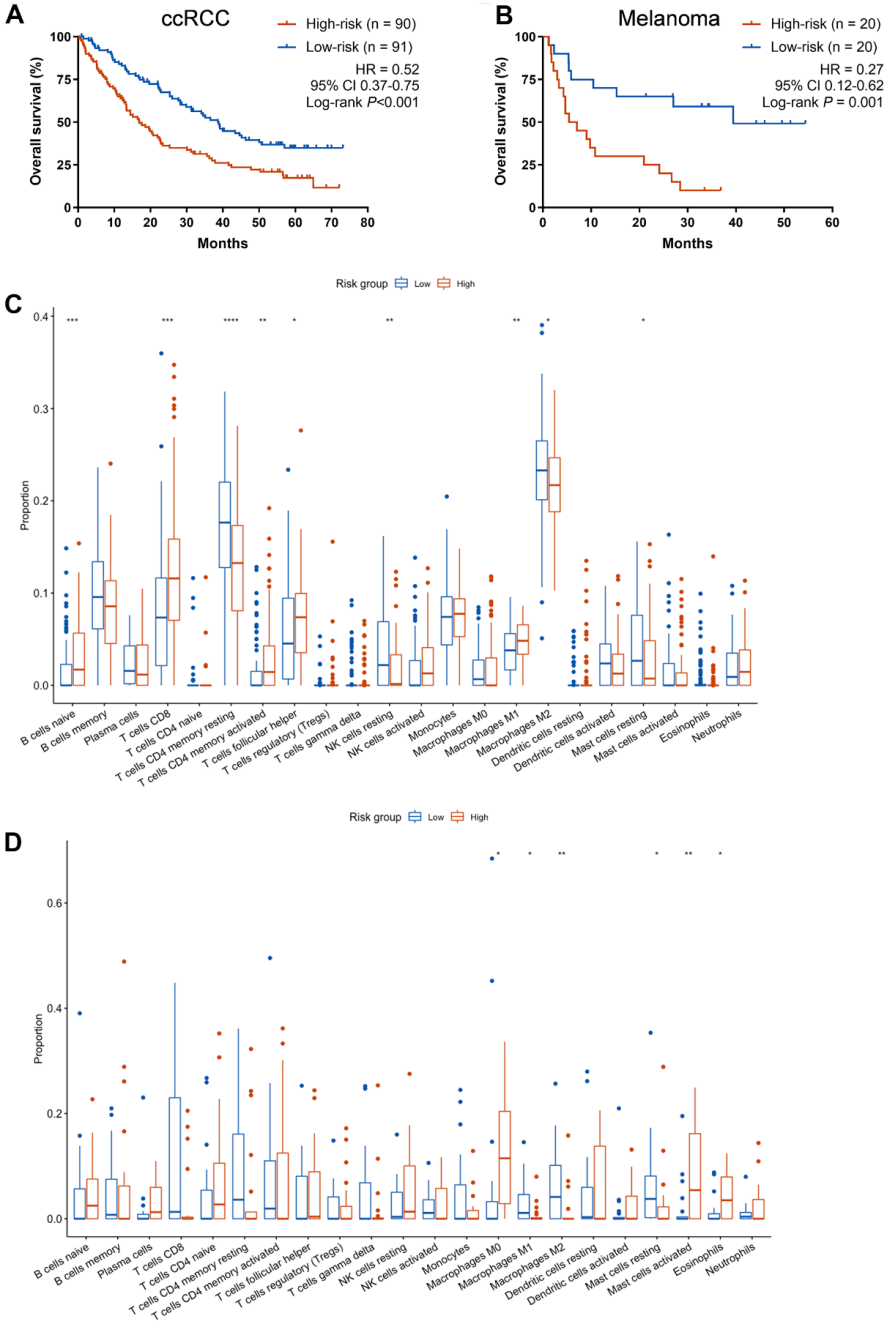
**Exploratory analysis of the CCCP risk score in ccRCC and melanoma cohort.** (**A**, **B**) Kaplan-Meier curves of OS comparing patients with high- and low-risk in the ccRCC cohort (**A**) and melanoma cohort (**B**) treated with ICIs. (**C**) Comparison of immune cell infiltrations between patients with high- and low- risk score in melanoma cohort. (**D**) Comparison of immune cell infiltrations between patients with high- and low- risk score in ccRCC cohort. CCCP, complement and coagulation cascades pathway; ccRCC, clear cell renal cell carcinoma; Tfh, T follicular helper cells.

In order to further explore the immunotherapeutic predictive utility of CCCP risk score in other tumors, ccRCC and melanoma cohorts with patients treated with ICIs and available mRNA expression data were exploratively analyzed. In the ccRCC cohort, patients with low-risk score showed better OS than those with high-risk score (median OS, 38.6 vs 16.9 months; HR, 0.52, 95% CI 0.37-0.75, P< 0.001, Figure 4A). Similar results were noted in the melanoma cohort (median OS, 39.5 vs 6.2 months; HR, 0.27, 95% CI 0.12-0.62, P = 0.001, Figure 4B). Besides, we found that the proportion of macrophages M0, activated mast cells, and Eosinophils were higher in the high-risk group, while macrophages M1 and M2, and resting mast cells were higher in the low-risk group in melanoma cohort (Figure 4C). In the ccRCC cohort, the proportion of naive B cells, CD8^+^ T cells, activated CD4^+^ memory T cells, Tfh cells, and macrophages M1 were higher in the high-risk group, while resting CD4^+^ T memory cells, resting NK cells, macrophages M2, and resting mast cells were higher in the low-risk group (Figure 4D). Altogether, these results suggested that the high-risk group exhibited an immunosuppressive TME. The CCCP risk score is of potential predictive utility across different cancer patients treated with ICIs.

## DISCUSSION

In the present study, 1,613 DEGs were firstly identified between responders and non-responders in the mUC cohort. The DEGs in CCCP were significantly enriched in the gene set of non-responders of ICIs. The CCCP risk score including *C2, C1QB, SERPING1, CFB, MASP1, F8*, and *F2R* genes was then trained and tested in the mUC cohort. The CCCP risk score could identify patients with ICIs who had better OS, while there was no significant association between the risk score and OS in the mUC patients without ICIs, suggesting rather a predictive than a prognostic role. In multivariable cox regression analysis, the CCCP risk score was associated with OS in mUC independent of PD-L1 expression and TMB. In addition, the activity of immune checkpoint, T cell receptor, and T-effector and IFN-γ pathway were significantly lower in high-risk group, suggesting a relative immunosuppressive TME. In exploratory analysis, the CCCP risk score could also predict the response to ICIs in ccRCC and melanoma.

An ideal predictive biomarker for immunotherapy is supposed to stratify patients who can benefit from ICIs, and meanwhile has no association with survival for patients receiving other treatments. In the present study, we first demonstrated that the CCCP risk score was associated with OS in the training and test sets from the mUC cohort treated with ICIs. Since ICIs are currently approved by FDA for advance-stage UC, we further selected stage IV patients who mainly accepted chemotherapy from TCGA-BLCA cohort and conducted the same analysis. There were no significant association between the CCCP risk score and the OS in TCGA-BLCA cohort, suggesting that CCCP risk score did not predict the benefit from chemotherapy in advanced BLCA. These results indicated that the CCCP risk score was a predictor but not prognostic indicator for mUC. However, the predictive effect of the CCCP risk score warranted further validation in randomized controlled trials.

Previous studies have shown that higher infiltrated M1 macrophages, activated NK cells, activated CD4 + memory T cells, and Tfh cells were associated with a significantly better prognosis [37–42]. We investigated the component of tumor infiltrating lymphocytes in mUC to explore the influence of complement system on TME. In the present study, the CCCP risk score was negatively related to the infiltration level of macrophages M1, activated NK cells, activated CD4^+^ memory T cells, and Tfh. Moreover, macrophages M1, activated NK cells, activated CD4^+^ memory T cells, and Tfh were significantly higher in the low-risk group. The fraction of naive CD4^+^ T cells and resting NK cells were significantly higher in the high-risk group and positively correlated with the risk score. Similarly, recent studies have revealed that CCCP have a multifaceted role in immune regulation and cancer [14, 16, 21, 43]. Complement acts as an immune surveillance against cancer by eliciting potent anti-tumor cytotoxic responses. In contrast, complement proteins, such as C3, C3a and C5a, downregulated the antitumor T cell responses through recruiting and activating MDSCs, macrophages M2, or T regulatory cells (Tregs) [11, 44–46]. Collectively, complement activation may shape an encouraging immune-enhanced microenvironment thus impacting the efficacy of ICIs in our study. However, mechanistic investigation including cell and molecular biology study for complement-mediated differentiation of immune cells is needed to further interpret these results.

In present study, we found that the CCCP risk score not only predicted the efficacy of ICIs in mUC, but also served as a predictor for ICIs in ccRCC and melanoma. To investigate the potential consistent association, we found that the infiltrations of macrophages M1 and M2 were both higher in the low-risk group. Macrophages M2 generally represents poor prognosis in melanoma according to the previous report [36], while increased proportion of macrophages M1 was associated with better prognosis in lung [25] and colorectal cancer [47]. The skewing of TAMs into M1 phenotype, may represent the better clinical prognosis. In terms of ccRCC, we observed CD8^+^ T cells were more abundant in the high-risk group, which was consistent with a previous study that a high density of CD8^+^ T cell was associated with poor survival in ccRCC [48]. Overall, the above results suggest that the CCCP risk score has potential to be a pan-cancer immunotherapeutic predictive biomarker, however, more evidences in other cancer types are warranted.

The development of biomarkers that predicts the efficacy of ICIs falls behind the amazing therapeutic innovation, except for PD-1 and TMB which have been used in clinical practice. Comprehensive and effective biomarkers are still under research. Liang et al., proposed a risk model based on the immune-related genes for predicting immunotherapeutic responses and identifying the patients who may benefit from ICIs in mUC [49]. In addition, a prognosis and predictive model has been constructed based on four hypoxia-related genes and verified its value in predicting benefit of ICIs in mUC [50]. Moreover, DNA damage response (DDR) pathway has been reported as a predictor for ICIs efficacy in mUC patients [51]. Though the importance of CCCP in TME has been broadly investigated [11, 14, 17], the predictive value of the CCCP in predicting ICIs benefit in mUC was seldom researched. To our knowledge, this is the first study regarding CCCP in predicting response to ICIs in Muc. The seven-gene signature in CCCP may represent a cost-effective method for further utility in clinic.

Several limitations should not be ignored. First, even though we utilized the machine learning approach to select the optimal candidate genes and a data-splitting strategy to ensure the robustness of the CCCP risk model, there is still a lack of independent validation cohort. Second, ccRCC and melanoma cohorts were tentatively included to test the predictive role of the CCCP risk score for ICIs regimen. Whether it could serve as a pan-cancer indicator need further validation. Third, the underlying mechanism between CCCP and immune environment needs to be further explored.

In conclusion, we established a CCCP risk score to predict the efficacy of ICIs in mUC patients. The patients with a low-risk score tended to have a better response to ICIs and a longer life time probably due to the immune-activated TME. In addition, CCCP may play a crucial role in T-effector, IFN-γ and T-cell receptor pathway. Future studies are needed to further validate the clinical utility of the CCCP risk score in the patients treated with ICIs in mUC and other cancer types.

### Nomenclature

ICIs, immune checkpoint inhibitors; mUC, metastatic urothelial cancer; PD-L1, programmed death-ligand 1 (PD-L1); ccRCC, clear cell renal cell carcinoma; UC, urothelial carcinoma; TMB, tumor mutational burden; CR, complete response; PR, partial response; SD, stable disease; PD, progressive disease; FDR, false discovery rate; DEGs, differentially expressed genes; OS, overall survival; DFS, disease-free survival; LASSO, least absolute shrinkage and selection operator; ssGSEA, single sample gene set enrichment analysis; ORR, objective response rate; HR, Hazard’s ratio; TME, tumor microenvironment; TILs, tumor infiltrating leukocytes.

## Supplementary Material

Supplementary Figures

Supplementary Tables 1 and 2

Supplementary Table 3

Supplementary Table 4
