# Supplementary material for: Complement and coagulation cascades pathway-related signature as a predictor of immunotherapy in metastatic urothelial cancer
Source: Aging (Albany NY). 2023 Sep 24;15(18):9479–98. doi: 10.18632/aging.205022 (PMC10564431; doi:10.18632/aging.205022)
Supplement: Supplementary Figures [file aging-15-205022-s001.pdf]

SUPPLEMENTARY FIGURES

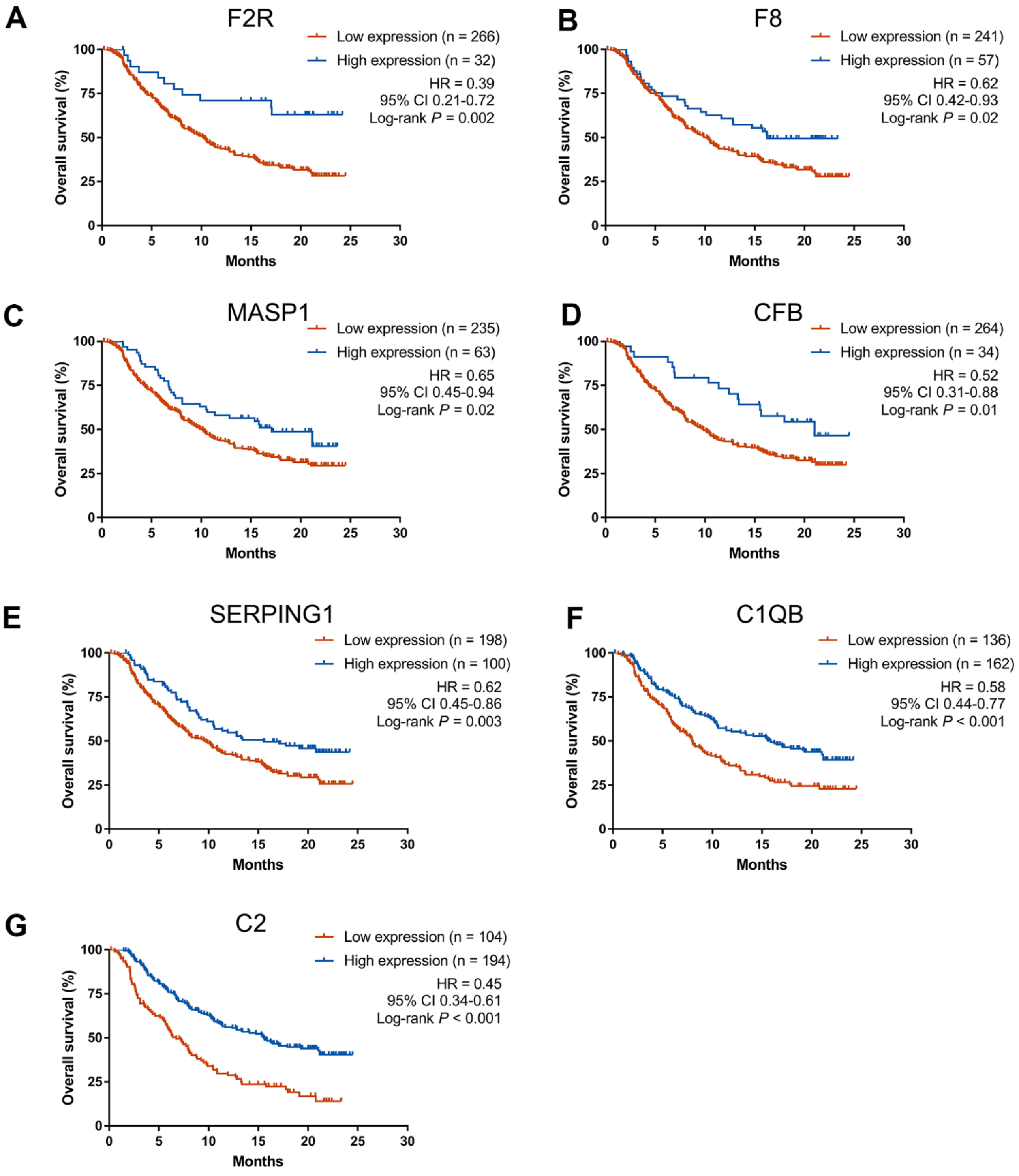

Supplementary Figure 1. Kaplan-Meier curves of OS comparing patients with high and low gene expression of the seven core genes included in the CCCP risk model: F2R (**A**), F8 (**B**), MASP1 (**C**), CFB (**D**), SERPING1 (**E**), C1QB (**F**), C2 (**G**).

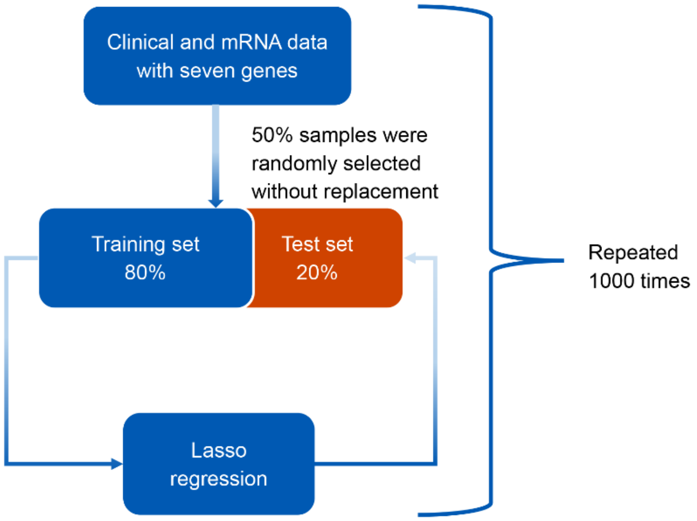

Supplementary Figure 2. The CCCP risk model was constructed with seven selected genes in complement and coagulation cascades pathway. The data-splitting strategy was implemented with 80% and 20% samples, which were used as training and test set respectively by repeating 1000 times.

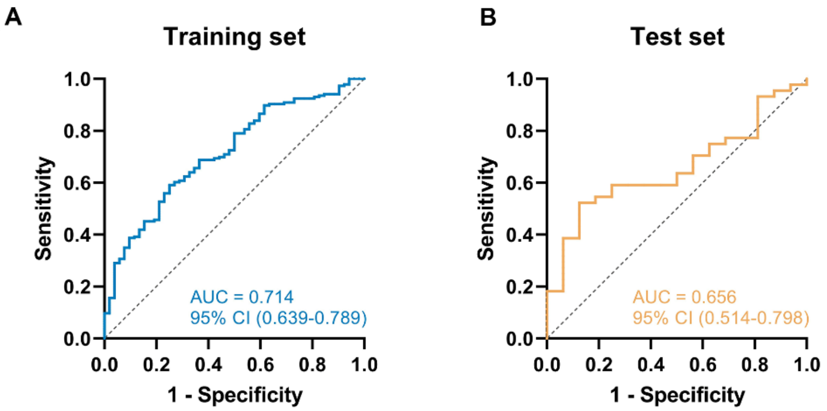

Supplementary Figure 3. The ROC of CCCP risk score in predicting response to ICIs in the training set (**A**) and test set (**B**).

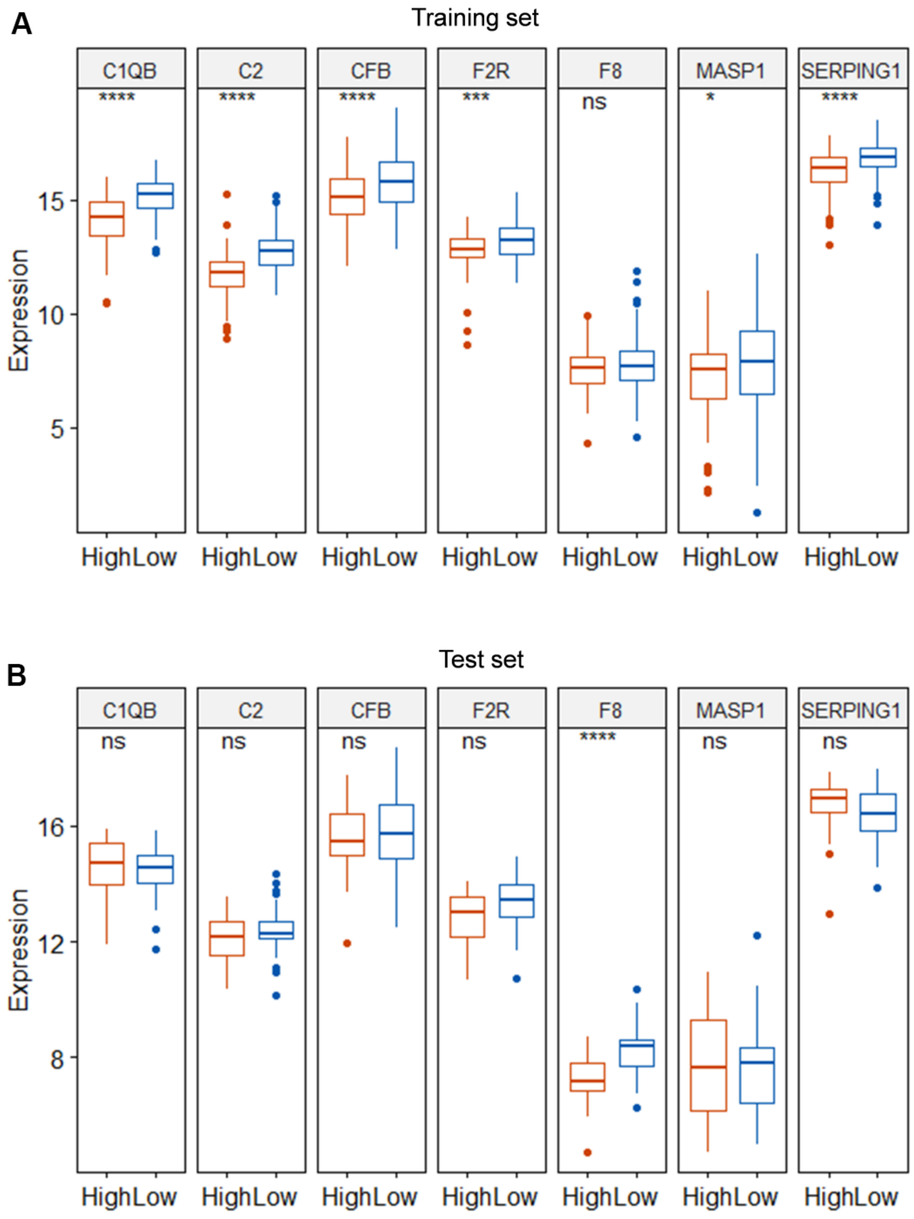

Supplementary Figure 4. Comparison of the expression of the seven core genes between patients with high- and low-risk in the training (**A**) and test (**B**) sets.

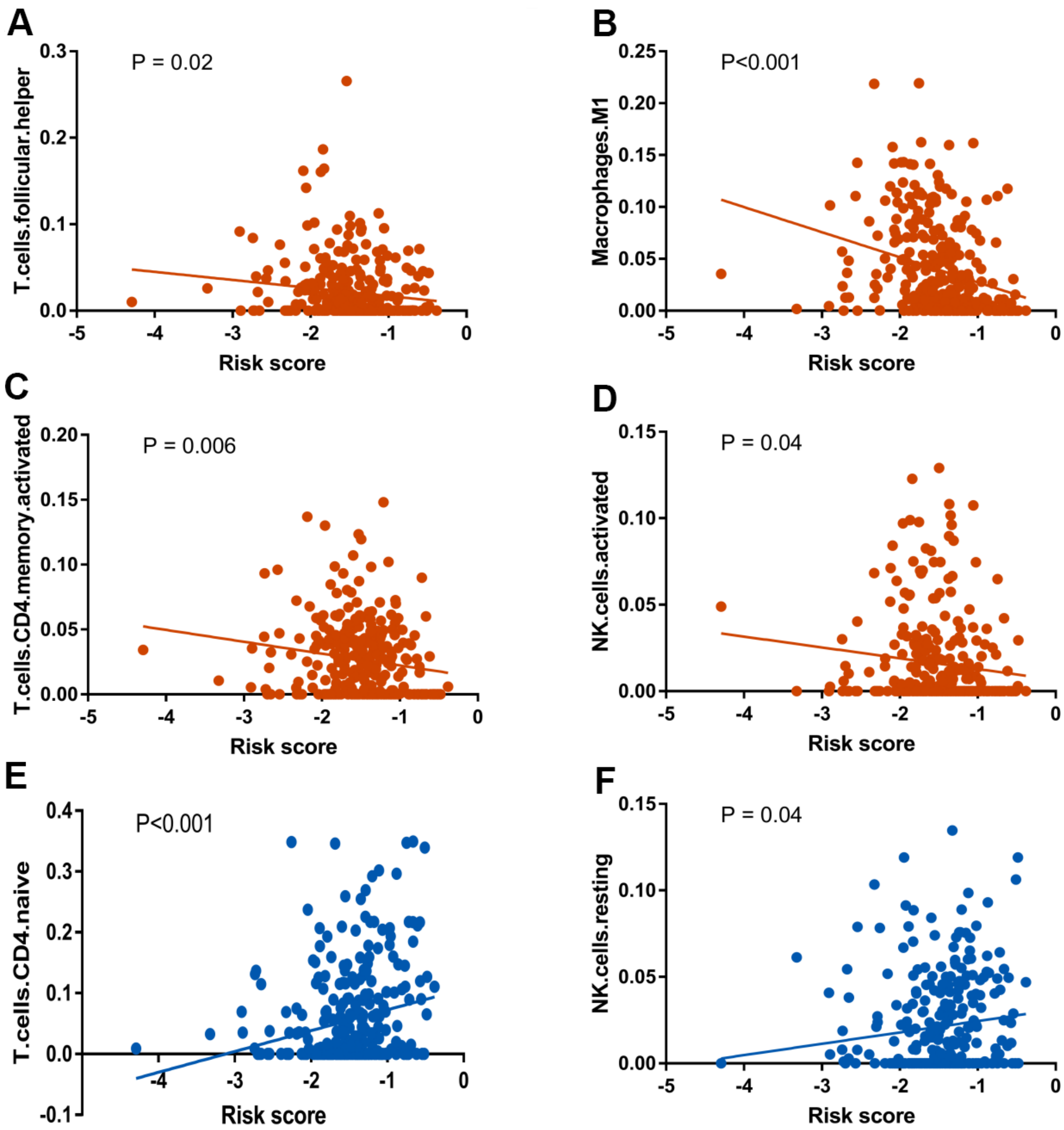

Supplementary Figure 5. Correlation between the risk score and follicular helper T cells (**A**), Macrophages M1 (**B**), activated CD4+ memory T cells (**C**), activated NK cells (**D**), naive CD4+ T cells (**E**), and resting NK cells (**F**). NK: natural killer.

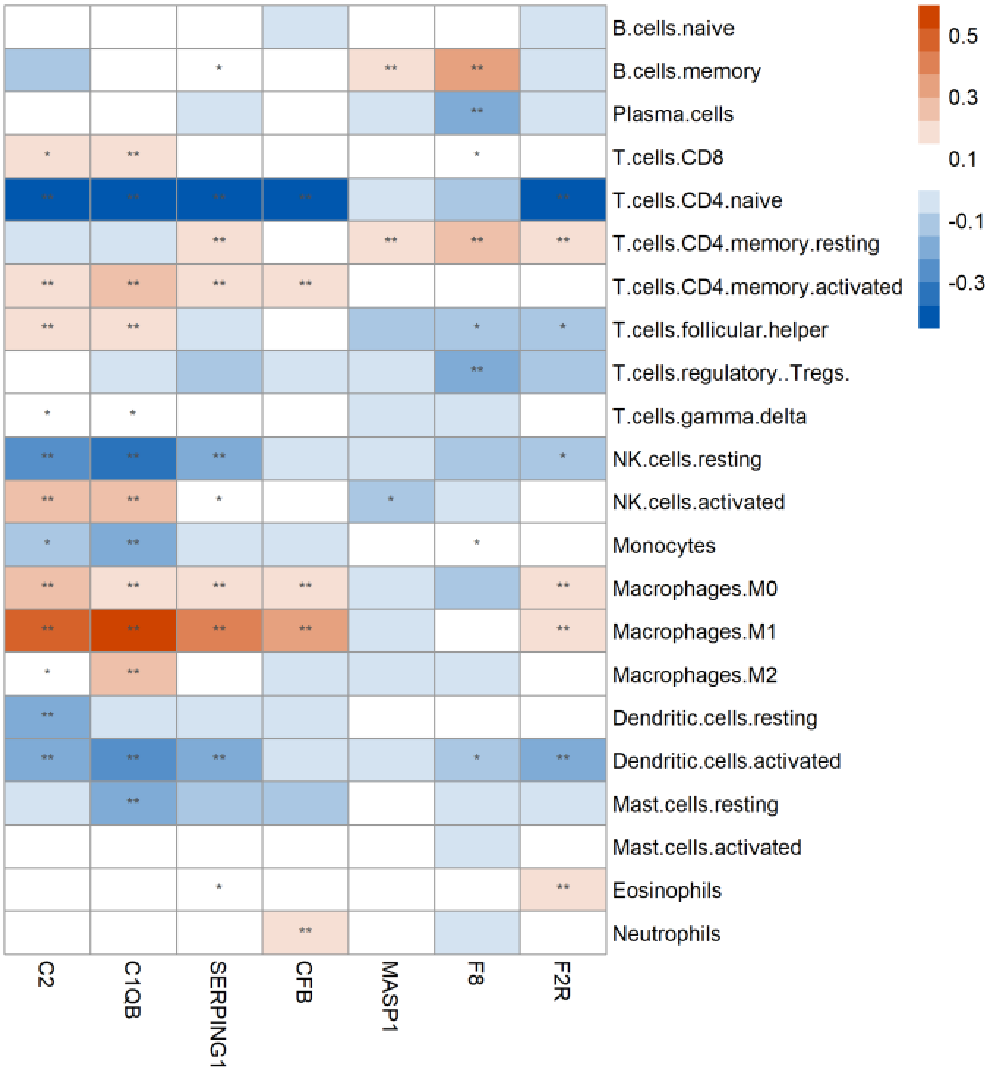

Supplementary Figure 6. Correlation between the immune cell infiltrates and the expression of the seven core genes included in the CCCP risk model.
