# Supplementary material for: Complement and coagulation cascades pathway-related signature as a predictor of immunotherapy in metastatic urothelial cancer
Source: Aging (Albany NY). 2023 Sep 24;15(18):9479–98. doi: 10.18632/aging.205022 (PMC10564431; doi:10.18632/aging.205022)
Supplement: Supplementary Tables 1 and 2 [file aging-15-205022-s002.pdf]

SUPPLEMENTARY TABLES

Supplementary Table 1. Baseline clinical characteristics of the patients.

Dataset	IMvigor210	ccRCC	Melanoma	TCGA-BLCA stage IV
Platform	Illumina HiSeq 2500	Illumina RNA-seq	Illumina RNA-seq	Illumina RNA-seq
No. of patients	298	181	40	131
Age, median (range)	NA	NA	60.5 (22-83)	69 (44-90)
Sex				
Male	233 (78%)	NA	26 (65%)	97 (74%)
Female	65 (22%)	NA	14 (35%)	34 (26%)
Anti-PD1/L1 regimen	Atezolizumab	Nivolumab	Ipilimumab	NA
Survival outcome	OS	OS, PFS	OS, DFS	OS, PFS

Abbreviations: ccRCC, clear cell renal cell carcinoma; NA, not available; OS, overall survival; DFS, disease-free survival.

Supplementary Table 2. The gene list of complement and coagulation cascades pathway.

Gene list of complement and coagulation cascades pathway
A2M, BDKRB1, BDKRB2, C1QA, C1QB, C1QC, C1R, C1S, C2, C3, C3AR1, C4A, C4B, C4BPA, C4BPB, C5, C5AR1, C6, C7, C8A, C8B, C8G, C9, CD46, CD55, CD59, CFB, CFD, CFH, CFI, CPB2, CR1, CR2, F10, F11, F12, F13A1, F13B, F2, F2R, F3, F5, F7, F8, F9, FGA, FGB, FGG, KLKB1, KNG1, MASP1, MASP2, MBL2, PLAT, PLAU, PLAUR, PLG, PROC, PROS1, SERPINA1, SERPINA5, SERPINC1, SERPIND1, SERPINE1, SERPINF2, SERPING1, TFPI, THBD, VWF
