# Supplementary material for: Complement and coagulation cascades pathway-related signature as a predictor of immunotherapy in metastatic urothelial cancer
Source: Aging (Albany NY). 2023 Sep 24;15(18):9479–98. doi: 10.18632/aging.205022 (PMC10564431; doi:10.18632/aging.205022)
Supplement: Supplementary Table 3 [file aging-15-205022-s003.docx]

**Supplementary Table 3. Summary results of KEGG enrichment analysis.**

| ID | Description | GeneRatio | BgRatio | pvalue | p.adjust | qvalue | geneID | Count |
| --- | --- | --- | --- | --- | --- | --- | --- | --- |
| hsa04610 | Complement and coagulation cascades | 37/632 | 85/8108 | 1.69E-19 | 5.08E-17 | 4.53E-17 | 10747/10877/1379/1380/2147/2153/2158/2159/2160/2243/2244/2266/3053/3075/3078/3080/3426/3827/462/5055/5104/5265/5340/5345/5648/7035/718/720/721/722/729/730/731/732/735/7448/81494 | 37 |
| hsa04080 | Neuroactive ligand-receptor interaction | 65/632 | 341/8108 | 5.33E-12 | 8.02E-10 | 7.15E-10 | 1081/10886/1132/1144/122042/1269/134864/1392/1394/151/1511/165829/1812/183/185/186/1902/2147/22953/2492/2554/2556/2559/2561/2565/2587/2642/2890/2892/2895/2898/2900/2903/2904/2905/2916/2922/3062/3350/3354/3356/3827/3952/4887/4922/4986/5024/5028/5340/5540/5618/5644/5645/5739/5745/59350/6344/6750/6865/6870/718/90226/9127/9248/9568 | 65 |
| hsa00830 | Retinol metabolism | 24/632 | 68/8108 | 9.37E-11 | 9.40E-09 | 8.38E-09 | 124/125/126/127/131/145226/1544/1548/1551/1559/1562/1576/1592/195814/29785/316/53630/54575/7363/7364/7365/7367/79799/8630 | 24 |
| hsa00982 | Drug metabolism - cytochrome P450 | 24/632 | 72/8108 | 3.67E-10 | 2.76E-08 | 2.46E-08 | 124/125/126/127/131/1544/1548/1557/1559/1571/1576/218/2328/27306/2938/2939/2949/316/54575/7363/7364/7365/7367/79799 | 24 |
| hsa04976 | Bile secretion | 25/632 | 89/8108 | 8.78E-09 | 5.29E-07 | 4.71E-07 | 10599/1080/10864/123264/1576/28234/361/3781/486/5244/54575/570/6344/64240/6523/6550/7363/7364/7365/7367/760/79799/8431/8647/9376 | 25 |
| hsa00980 | Metabolism of xenobiotics by cytochrome P450 | 23/632 | 78/8108 | 1.26E-08 | 6.30E-07 | 5.62E-07 | 124/125/126/127/131/1544/1548/1559/1571/1576/1645/218/27306/2938/2939/2949/29785/54575/7363/7364/7365/7367/79799 | 23 |
| hsa00140 | Steroid hormone biosynthesis | 18/632 | 61/8108 | 4.82E-07 | 2.07E-05 | 1.85E-05 | 1544/1551/1571/1576/1586/1588/1589/1645/3292/412/54575/6716/7363/7364/7365/7367/79799/8630 | 18 |
| hsa00591 | Linoleic acid metabolism | 12/632 | 29/8108 | 6.84E-07 | 2.55E-05 | 2.27E-05 | 100137049/123745/1544/1557/1559/1571/1576/50487/5319/5320/5322/84647 | 12 |
| hsa05204 | Chemical carcinogenesis - DNA adducts | 19/632 | 69/8108 | 7.62E-07 | 2.55E-05 | 2.27E-05 | 1544/1548/1551/1557/1559/1562/1571/1576/27306/2938/2939/2949/54575/5743/7363/7364/7365/7367/79799 | 19 |
| hsa04978 | Mineral absorption | 17/632 | 60/8108 | 1.88E-06 | 5.67E-05 | 5.06E-05 | 10568/115019/140803/341208/4489/4493/4495/4496/4499/4501/486/492/6523/6543/6550/6569/7018 | 17 |
| hsa04060 | Cytokine-cytokine receptor interaction | 45/632 | 295/8108 | 8.31E-06 | 0.000227453 | 0.000202834 | 10563/1235/1271/1437/1440/2662/268/27179/2919/338376/3442/3458/353500/3558/3569/3576/3589/3598/3627/3952/3976/4050/4283/4804/4982/53833/5473/5618/56300/59067/6352/6357/6358/6360/6369/6372/6373/643/7066/7850/8200/8600/8740/9173/959 | 45 |
| hsa00350 | Tyrosine metabolism | 12/632 | 36/8108 | 9.92E-06 | 0.000248704 | 0.000221785 | 124/125/126/127/131/1644/218/3081/316/3242/5409/6898 | 12 |
| hsa04979 | Cholesterol metabolism | 14/632 | 50/8108 | 1.77E-05 | 0.000410783 | 0.000366322 | 27329/335/336/337/345/350/4018/4023/4036/55908/64240/6770/8435/8647 | 14 |
| hsa00590 | Arachidonic acid metabolism | 15/632 | 61/8108 | 4.84E-05 | 0.001040606 | 0.000927976 | 100137049/123745/1557/1559/1571/2687/27306/2878/50487/5319/5320/5322/5742/5743/84647 | 15 |
| hsa04950 | Maturity onset diabetes of the young | 9/632 | 26/8108 | 9.36E-05 | 0.001803273 | 0.001608094 | 3170/3172/3651/4760/4821/5080/5313/6514/6927 | 9 |
| hsa05146 | Amoebiasis | 20/632 | 102/8108 | 9.59E-05 | 0.001803273 | 0.001608094 | 1437/1511/2774/2919/338382/3458/3569/3576/383/4583/5273/5275/6317/6318/731/732/735/7850/909/911 | 20 |
| hsa05150 | Staphylococcus aureus infection | 19/632 | 96/8108 | 0.000125128 | 0.002215502 | 0.001975705 | 10747/1668/1828/2204/2266/3075/342574/3426/3861/3866/3868/3881/3884/390792/5340/5648/718/720/721 | 19 |
| hsa05033 | Nicotine addiction | 11/632 | 40/8108 | 0.000168605 | 0.002819458 | 0.002514292 | 246213/2554/2556/2559/2561/2565/2890/2892/2903/2904/2905 | 11 |
| hsa04726 | Serotonergic synapse | 21/632 | 115/8108 | 0.000186792 | 0.002959181 | 0.002638892 | 100137049/10411/121278/123745/1557/1559/1562/1644/170572/2561/260293/2786/3350/3354/3356/3781/5742/5743/6532/6571/779 | 21 |
| hsa04061 | Viral protein interaction with cytokine and cytokine receptor | 19/632 | 100/8108 | 0.000219513 | 0.003193092 | 0.002847485 | 10563/1235/2919/3558/3569/3576/3627/4283/53833/5473/6352/6357/6358/6360/6369/6372/6373/643/8740 | 19 |
| hsa04614 | Renin-angiotensin system | 8/632 | 23/8108 | 0.000222774 | 0.003193092 | 0.002847485 | 1215/1359/1511/183/185/186/3816/59272 | 8 |
| hsa04974 | Protein digestion and absorption | 19/632 | 103/8108 | 0.00032656 | 0.004467938 | 0.003984347 | 1280/1294/1295/1300/1302/1308/1359/136227/153201/486/5644/5645/59272/6519/6543/6550/7373/7512/91522 | 19 |
| hsa04742 | Taste transduction | 16/632 | 86/8108 | 0.000859953 | 0.011203682 | 0.009991044 | 170572/22953/2554/2556/2559/259291/3350/3354/5024/5028/5136/6326/6338/80835/9033/9568 | 16 |
| hsa00040 | Pentose and glucuronate interconversions | 9/632 | 34/8108 | 0.000893317 | 0.011203682 | 0.009991044 | 231/54575/57016/7363/7364/7365/7367/79799/9365 | 9 |
| hsa00983 | Drug metabolism - other enzymes | 15/632 | 80/8108 | 0.001137412 | 0.013694439 | 0.012212212 | 1066/1548/1571/1576/1807/2938/2939/2949/54575/6241/7363/7364/7365/7367/79799 | 15 |
| hsa00360 | Phenylalanine metabolism | 6/632 | 17/8108 | 0.001285127 | 0.014877818 | 0.013267507 | 10249/1644/218/3242/5053/6898 | 6 |
| hsa04975 | Fat digestion and absorption | 10/632 | 43/8108 | 0.001395863 | 0.015561291 | 0.013877003 | 2168/29881/335/337/50487/5319/5320/5322/64240/84647 | 10 |
| hsa00592 | alpha-Linolenic acid metabolism | 7/632 | 25/8108 | 0.002345104 | 0.025209866 | 0.022481259 | 100137049/123745/50487/5319/5320/5322/84647 | 7 |
