# Supplementary material for: Complement and coagulation cascades pathway-related signature as a predictor of immunotherapy in metastatic urothelial cancer
Source: Aging (Albany NY). 2023 Sep 24;15(18):9479–98. doi: 10.18632/aging.205022 (PMC10564431; doi:10.18632/aging.205022)
Supplement: Supplementary Table 4 [file aging-15-205022-s004.pdf]

Supplementary Table 4. Summary results of LASSO regression.

	Training subset	Test subset
Not significant	0	143 (14.3%)
Significant	1000 (100%)	857 (85.7%)
